# Do ABO Blood Group Antigens Hamper the Therapeutic Efficacy of Mesenchymal Stromal Cells?

**DOI:** 10.1371/journal.pone.0085040

**Published:** 2014-01-13

**Authors:** Guido Moll, Annika Hult, Lena von Bahr, Jessica J. Alm, Nina Heldring, Osama A. Hamad, Lillemor Stenbeck-Funke, Stella Larsson, Yuji Teramura, Helene Roelofs, Bo Nilsson, Willem E. Fibbe, Martin L. Olsson, Katarina Le Blanc

**Affiliations:** 1 Division of Clinical Immunology and Transfusion Medicine, Department of Laboratory Medicine, Karolinska Institutet, Stockholm, Sweden; 2 Division of Hematology and Transfusion Medicine, Department of Laboratory Medicine, Lund University, Lund, Sweden; 3 Hematology Center, Karolinska University Hospital Huddinge, Stockholm, Sweden; 4 Rudbeck Laboratory, Department of Immunology, Genetics and Pathology, Uppsala University, Sweden; 5 Department of Immunohematology and Blood Transfusion, Leiden University Medical Center, Leiden, The Netherlands; Rutgers - New Jersey Medical School, United States of America

## Abstract

Investigation into predictors for treatment outcome is essential to improve the clinical efficacy of therapeutic multipotent mesenchymal stromal cells (MSCs). We therefore studied the possible harmful impact of immunogenic ABO blood groups antigens – genetically governed antigenic determinants – at all given steps of MSC-therapy, from cell isolation and preparation for clinical use, to final recipient outcome.

We found that clinical MSCs do not inherently express or upregulate ABO blood group antigens after inflammatory challenge or *in vitro* differentiation. Although antigen adsorption from standard culture supplements was minimal, MSCs adsorbed small quantities of ABO antigen from fresh human AB plasma (ABP), dependent on antigen concentration and adsorption time. Compared to cells washed in non-immunogenic human serum albumin (HSA), MSCs washed with ABP elicited stronger blood responses after exposure to blood from healthy O donors in vitro, containing high titers of ABO antibodies. Clinical evaluation of hematopoietic stem cell transplant (HSCT) recipients found only very low titers of anti-A/B agglutination in these strongly immunocompromised patients at the time of MSC treatment. Patient analysis revealed a trend for lower clinical response in blood group O recipients treated with ABP-exposed MSC products, but not with HSA-exposed products.

We conclude, that clinical grade MSCs are ABO-neutral, but the ABP used for washing and infusion of MSCs can contaminate the cells with immunogenic ABO substance and should therefore be substituted by non-immunogenic HSA, particularly when cells are given to immunocompentent individuals.

## Introduction

MSCs are tested in a large number of clinical trials with focus on exploiting their regenerative and immune modulatory properties [1]–[3]. The treatment is safe [3], [4], but efficacy of the first generation product is low [3], with an average clinical response rate of 68% at its latest follow up [5]. We have worked on identifying potential predictors of improved outcome, such as better patient stratification, use of early passage cells [6], and general improvements in blood compatibility of the product [7]. We here study the possible impact of immunogenic ABO antigens on the outcome of MSC therapy, from *ex vivo* preparation, to cell infusion, and consecutive patient response evaluation.

The ABO blood group is one of the major immunogenic barriers hampering tissue transplantation into immunocompentent hosts [8]. ABO donor blood groups are therefore readily available from hospital routine assessment without increased costs. The clinical MSCs could either display intrinsic ABO antigen expression according to their genetic determinants, or be externally contaminated. Carbohydrate blood groups are not encoded by genes directly, but blood group genes encode glycosyltransferases that synthesize the oligosaccharide epitopes [9]. Thus A/B antigens are added to the H core structure by A/B glycosyltransferases, encoded by the *ABO* gene. Methylation of the *ABO* proximal promoter is associated with down-regulation of A/B transcripts in hematological malignancy [10], and down-regulation can also be found in tumors compared to normal tissue [11]. Transient depression in A antigen expression has also been observed in pregnancy [12]. The specific reasons for conditional *ABO* promoter methylation remain elusive, but down-modulation of blood group antigens appears to be associated with classical states/sites of immune privilege [9].

Clinical MSCs could also be contaminated with ABO antigens from culture supplements, and washing buffers, used for cell infusion. Antigens showing cross-reactivity with ABO antibodies are potentially found in animal components [8], such as fetal calf serum (FCS) [13], and its human substitutes, such as pooled human AB serum (ABS) and platelet rich plasma (PRP) [14]–[17]. Prior studies indicated that ABO antigens are not expressed in native or *in vitro* differentiated MSCs and not adsorbed from culture medium [18], [19]. Antigen adsorption from AB plasma (ABP), which is often used for washing and infusion of MSCs, was not investigated. Prior studies further suggested human serum albumin (HSA) as a non-immunogenic supplement [20]. Differences in antigenicity exist for human donor sera/plasmas with respect to variation in antibody and soluble antigen content, and secretor status [9]. Classically, human blood group AB serum/plasma is considered to be the most reasonable choice for avoiding harmful reactions, due to its lack of ABO antibodies [16]. However, highly immunogenic soluble A and B antigens can be present [9], which could potentially be adsorbed to clinical grade MSCs. Transplant patients receiving ABO-incompatible grafts may show three types of immune response to incompatible A/B antigens: 1) Rejection, 2) Accommodation, and 3) Tolerance [8]. It is unclear whether preformed naturally-occurring antibodies can impair the therapeutic efficiency of MSCs. In solid organ transplantation it was documented that particularly adult blood group O recipients constitute a risk group for early allograft rejection, due to higher titers of anti-A/B immunoglobulin G (IgG) antibodies [21].

Here, we have investigated the ABO-related therapeutic efficiency of MSCs, totaling a number of more than 100 cell infusions at our two centers. We first characterized the ABO-antigen status of the native therapeutic product and then analyzed the therapeutic outcome with respect to donor – recipient ABO status.

## Materials and Methods

### MSC Recipients and ethics statement

HSCT recipients from the Karolinska University Hospital (n = 48) and Leiden University Hospital (n = 25), who received treatment with MSCs between 2002 and 2011, were included in the analysis. The patients received myeloablative (n = 40) or reduced intensity (n = 33) conditioning and routine graft-versus-host disease (GvHD) prophylaxis according to previously published procedures and patient outcome has been included in previous reports [5], [6], [22]. The patients received 1–5 infusions of MSCs, (median 1 infusion) and 105 infusions were included in the analysis. All response data are presented as per infusion. The indications for MSC treatment were acute GvHD (83 infusions) or hemorrhagic cystitis (22 infusions). The MSCs were obtained from HLA-identical siblings (n = 3), haploidentical donors (n = 12) or 3^rd^ party unrelated donors (n = 90). They were given in passage 1 – 4 (median 3) and in a median dose of 1.7×10^6^ cells/kg (range 0.7 – 4.2×10^6^ cells/kg). For rapid availability, most of the cells were stored in liquid nitrogen and freshly thawed for IV infusion (n = 96) and some patients received one infusion each of freshly detached cells (n = 9). Donors and patients or their legal guardians gave written informed consent and the review board at Karolinska University Hospital Huddinge approved the study. All clinical research was conducted according to the principles expressed in the Declaration of Helsinki.

### ABO blood group typing of patients and ABO antibody titer in patient blood

ABO grouping including detection of anti-A and anti-B titers in patient blood was done by automated serological tests at the hospital site based on the micro-column technique and by agglutination of erythrocytes of known blood group.

### Isolation and preparation of therapeutic MSCs

To isolate MSCs, bone marrow aspirates of approximately 50 ml were taken from the iliac crest of healthy donors (n = 50; median age 37; range 6 – 68 years). The expansion and characterization of the MSCs was performed according to the guidelines of the MSC Consortium of the European Blood and Marrow Transplantation Group (EBMT) and approved by the Swedish National Board of Health and Welfare, as previously described in detail [5], [23], [24]. Briefly, bone marrow mononuclear cells were separated over a gradient of Redigrad (GE Health Care, Sweden), washed and resuspended in DMEM low-glucose medium (DMEM-LG; Invitrogen, Grand Island, NY); supplemented with 100 IU/ml penicillin, 0.1 mg/ml streptomycin, and 10% FCS (Hyclone, Logan, Utah, USA), or alternatively 5% human ABS or PRP, and plated at 1.6×10^5 ^cells/cm^2^. When the cultures approached confluence (>80%), the cells were detached by treatment with trypsin and EDTA (Invitrogen, Grand Island, NY) and replated/passaged at a density of 4.0×10^3^ cells/cm^2^ for up to four passages. Flow cytometry analysis indicated that the MSCs were positive for CD73, CD90 and CD105 but negative for CD14, CD31, CD34 and CD45. Adipogenic and osteogenic differentiation was evaluated as previously described [23]. The MSC suspensions were culture-negative for bacteria and fungi and polymerase chain reaction (PCR)-negative for *Mycoplasma* [5], [24].

### *ABO* genotype, promoter methylation, and transcript analysis on clinical MSCs

***ABO*** **and** ***FUT2*** **genotyping.** Two independent methods for determination of *ABO* genotyping were performed on DNA prepared from thawed clinical grade MSCs at the Nordic Reference Laboratory for Genetic Blood Group Typing at the University and Regional Laboratories in Lund, Sweden. These included an allele-specific primer (ASP) PCR [25] and a restriction fragment length polymorphism (RFLP) PCR [26] *FUT2* genotyping was performed with an unpublished method developed in house, assessing nucleotide position 428 (G/A) to determine secretor *vs*. non-secretor status.

***ABO*** **promoter methylation status.** The promoter methylation status was analyzed with MagMeDIP Kit (mc-magme-048; Diagenode, Belgium) according to manual. Briefly, DNA was isolated from MSCs (n = 4) and 1 μg sonicated DNA was used. Methylated DNA positive control and unmethylated DNA negative control were spiked into one sample in order to control for the assay. Sonicated DNA was added to two tubes: input DNA (10% of sonicated DNA) and immunoprecipitation (IP) sample DNA. IP incubation mix was added to both tubes, heated at 95°C for three minutes and briefly centrifuged at 4°C. The 10% input DNA samples were then stored at 4°C. The provided anti-5mC antibody was diluted 1:2 in water before making the antibody mix with MagBuffer A and C and added to the IP tubes. Magbeads (11 μl/IP) were washed and resuspended in bead wash buffer before added to the IP’s. The samples were incubated at 4°C over night. The DNA samples were washed three times with 100 µl ice-cold MagWash Buffer-1 and ice-cold MagWash Buffer-2. After the last wash, the last traces of Wash buffer were discarded. The bead pellets were kept on ice. Input samples were centrifuged briefly and from this point on treated in parallel with IP samples. DNA isolation buffer (DIB) was prepared adding 1 µL of Proteinase K per 100 µL of DIB. The tubes were removed from the Magnetic Rack and complete DIB was added to each sample and incubated at 55°C for 15 min and then at 100°C for 15 min. The samples were then centrifuged at 14,000 rpm for 5 min at 4°C and supernatants analyzed in a SYBR green Q-PCR assay using *ABO* promoter-specific primers: FW; CCCTTGACACCCTGTCTCC REV; AGCTTCACGGGTTCGTCTC. Primers for the methylated promoter (TSH2B) and the unmethylated promoter (GAPDH) were provided in the kit.

***ABO*** **transcript analysis.** To study mRNA transcripts in resting or stimulated cells, MSCs were subjected to standard differentiation or pro-inflammatory mediators. Adipogenic and osteogenic differentiation was induced [23], and confirmed by lipid vacuole formation/upregulation of gene aP2, and matrix mineralization, respectively. To activate MSCs with cytokines, the cells were exposed for 5 days either to 100 U/ml recombinant human interferon gamma (IFNg; Sigma Aldrich Ltd, UK), or to pro-inflammatory mediators secreted by activated PBMCs in trans-well mixed lymphocyte reactions (MLRs), and harvested for PCR analysis as described earlier [7]. Cell lysates were harvested with RLT buffer (Qiagen, Hilden, Germany), RNA was extracted using a Qiagen RNeasy minikit, and stored in RNAse-free water at −80 °C. Concentration and purity of RNA was estimated by reading absorbance at 260 and 280 nm with a spectrophotometer (Nanodrop; Thermo Fisher, Wilmington, DE). The cDNA samples used for PCR analysis were obtained with the high-capacity cDNA Reverse Transcription Kit (Applied Biosystems, Foster City, CA). QRT-PCR analysis was performed with the Applied Biosystems 7900 HT sequence detection system. The expression of beta-actin served as an internal standard: Ct  =  Ct_(Gene)_ − Ct_(Actin)_, with Ct being the cycle threshold of beta-actin or the gene of interest Results were calibrated against a negative control and further analyzed by the 2^−▵Ct^ method [7], [27].

### Flow cytometry detection of ABH antigens on MSCs cultured with different supplements and antigen adsorption from human AB plasma

A highly sensitive flow cytometry assay for detection of low levels of ABH histo-blood group antigens, previously developed for quality control of blood group A, B and AB red blood cells (RBCs) converted to type as group O was applied [28]. Its sensitivity was confirmed by detection of A antigen on RBCs of the weakly expressing, inherited A_x_ subgroup and also detection of the minute levels of A antigens present on group B RBCs. Paragloboside carbohydrate antigen served as positive control for secondary antibody binding, blood group O MSCs served as negative controls for anti-A and -B detection, and MSCs labeled with secondary antibody only were used as baseline values. To detect antigen adsorption from culture supplements, cells were isolated and expanded in the presence of 10% FCS, 5% ABS, or 5% PRP, as described above. Additionally, adsorption of blood group A/B-antigen from fresh clinical ABP, used as supplement for washing, reconstitution and clinical infusion of MSCs, was tested. Clinical MSC preparations were thawed, briefly washed in buffer containing 10% O plasma to remove DMSO from cryo-medium, then resuspended in a small volume of PBS, and aliquoted in FACS tubes for incubation with different types of plasmas for either 1 or 3 hours. As positive control for A/B-antigen adsorption MSCs were incubated with 100% A_1_B, and A_2_B plasma and as a negative control with 100% O non-secretor plasma (200.000 MSCs were incubated with 0.5 ml of undiluted plasma or with 2 ml PBS containing 10% plasma). Anti-A/B detection was performed on washed cells as described above. Additionally, MSCs were labeled with the PE-conjugated isotype control and positive (CD105-PE) or negative (CD14-PE) control antibodies. All antibodies are listed in Table S1.

### Whole blood exposure of fresh and thawed MSCs washed and resuspended in buffer containing either 10% ABP or HSA

Fresh or thawed MSCs were washed and resuspended in buffer containing either 10% ABP or HSA and exposed to blood by using the Chandler loop system, consisting of tubing with a heparinized inner surface (Corline Systems), as described previously [7], [29]–[31]. Fresh non-anti-coagulated blood group O donor blood was obtained from healthy volunteers who had given informed consent in accordance with the Helsinki Protocol and had received no medication for at least 10 days [7]. Briefly, pieces of tubing containing 7 ml of blood were prepared and supplemented with 100 µl of PBS containing 10% ABP or HSA +/– freshly detached or freeze-thawed MSCs. To determine the time course of reaction between blood and cells 1-ml samples from each blood tube were collected before cell addition and at 5, 15, 30, and 60 min after cell addition. Reactions were stopped by the addition of EDTA (pH 7.4, 10 mM final). Platelet and cell counts were obtained by using a cell counter (Beckman Coulter). The remaining sample volume was centrifuged at 3000 g for 20 min at 4°C. Plasma was collected, stored at −80°C, and formation of thrombin-anti-thrombin complex (TAT) and complement C3 activation fragment a (C3a) was measured by ELISA [7].

### Statistical analysis

Statistical analyses were performed using ANOVA and Student’s t-test. If the data did not fit a normal distribution, the Mann-Whitney or the Wilcoxon matched-pairs tests were used (two-tailed confidence intervals, 95%; P<0.05 was considered statistically significant; Prism 5.0; Graphpad Software). For clinical response, differences between responders and non-responders were evaluated using Fisher’s exact test, or when appropriate, Chi2-test.

## Results

### Native clinical MSCs do not express or upregulate ABO blood group antigens

**Figure 1:**
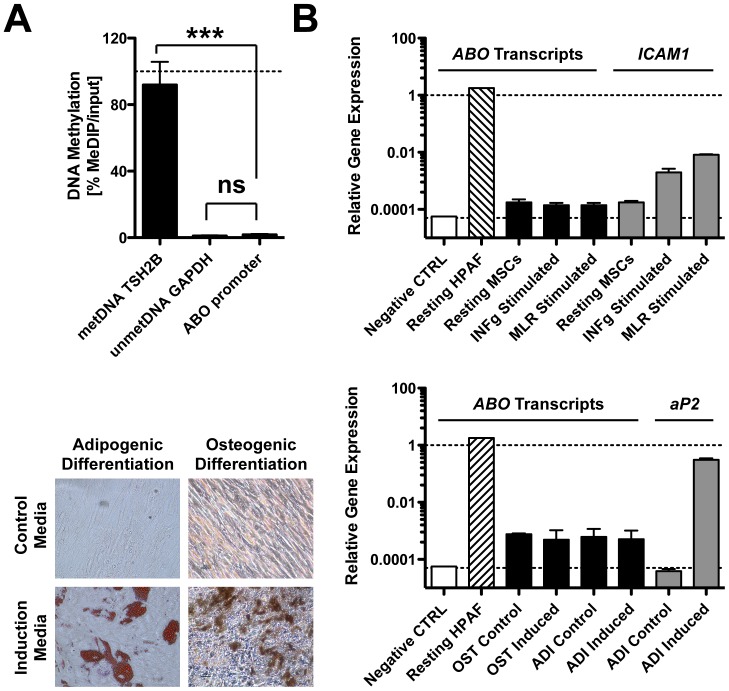
*ABO* promoter methylation and *ABO* transcript expression analysis. The *ABO* promoter methylation status (n = 4 clinical grade MSCs, 2 samples each) and the expression of ABO blood group gene transcripts in clinical grade MSCs (n = 2) of known blood group and secretor genotype (K16-*A^1^/A^1^-Se/se*, K25-*B/O^1^-Se/Se*) was tested with MagMeDIP Kit and quantitative real time PCR (qPCR), respectively. (**A**) DNA methylation (%, metDIP/input), of methylated *TSH2B*, unmethylated *GAPDH*, and *ABO* proximal promoter region. (**B**) *ABO* transcript analysis (qPCR) on resting MSCs compared to cells subjected to different types of induction treatments. MSCs were either stimulated for 5 days with 5 ng/ml of interferon-gamma (INFg), or activated for 5 days by inflammatory mediators released through a cell-impermeable membrane in trans-well mixed lymphocyte reactions (MLRs). MSCs were also subjected to 14-day *in vitro* differentiation with adipogenic (ADI) and osteogenic (OST) induction medium, or respective control medium. Adenocarcinoma cell line HPAF-II was used as positive control for *ABO* transcript expression and distilled H_2_O served as negative control. Control genes *ICAM1* and *aP2* served as positive controls for cytokine activation and adipogenic induction, respectively. Relative gene expression is shown compared to control gene beta-actin. Adipogenic and osteogenic differentiation was also confirmed with Oil red O staining for lipid rich vacuoles and von Kossa staining for mineralized matrix, respectively. Mean ± SD, *** *P* < 0.001.

The *ABO* and *FUT2* (secretor) genotypes of 26 clinical MSCs (cultured with 10% FCS) of unknown blood group were determined and their ABH histo-blood group phenotypes predicted (Table 1) [25], [26]. We hypothesized that secretor status may play a role in ABH expression and therefore typed for the 428G vs. A polymorphism in the *FUT2* gene, associated with secretor vs. non-secretor status, respectively. We found that 85% of tested MSCs had the secretor genotype, i.e. had the genotypes 428G/G or 428G/A. Flow cytometry testing with monoclonal anti-A, -B, and -H confirmed our earlier results [18], that A and B antigens are not expressed on the surface of freshly thawed clinical grade MSCs (Figure S1A), irrespective of their donor secretor status (Figure S1B). Weak positivity was found for the H antigen and strong positivity for the paragloboside positive control (not shown), confirming that these carbohydrate antigens are detectable even after trypsin treatment. We wondered if transcription of the *ABO* promoter region is silenced in MSCs by DNA methylation (Figure 1A) and found that the promoter is methylated to a similarly low degree as housekeeping gene GAPDH (P<0.001; GAPDH 5% vs. met-TSH2B 90%). We tested if stimulation of MSCs could induce expression of *ABO* transcripts in selected cells with known *ABO* and secretor genotype (K16-*A^1^*/*A^1^*-*Se*/*se* and K25-*B*/*O^1^*-*Se*/*Se*). None of the treatments (INF-gamma, MLRs, and adipogenic or osteogenic induction) resulted in up-regulation of *ABO* transcripts (Figure 1B), although the MSCs responded appropriately with up-regulation of control genes *ICAM1*, *aP2*, formation of lipid vacuoles, and matrix mineralization, respectively. As a positive control, we included the pancreatic adenocarcinoma cell line HPAF-II (blood group A Se/Se), which showed high levels of *ABO* transcripts comparable to control gene *GAPDH*.

**Table 1:** *ABO* genotyping and detection of blood group antigens on MSCs.

MSC ID	ABO-ASP Genotype	FUT2-ASP Genotype	Predicted Phenotype	anti-A [RFI]	anti-B [RFI]	anti-H [RFI]
**K09**	*A^1^O^1^*	nt. 428A/A (se/se)	A_1_ non-secretor	1.12	1.03	1.03
**K23**	*A^1^A^1^*	nt. 428A/A (se/se)	A_1_ non-secretor	1.12	1.04	6.06
**K01**	*A^1^O^1^*	nt. 428G/A (Se/se)	A_1_ secretor	0.99	0.95	4.97
**K07**	*A^1^A^1^*	nt. 428G/A (Se/se)	A_1_ secretor	1.08	0.91	1.94
**K14**	*A^1^O^1^*	nt. 428G/A (Se/se)	A_1_ secretor	1.01	0.99	1.63
**K16**	*A^1^A^1^*	nt. 428G/A (Se/se)	A_1_ secretor	1.57	1.03	1.25
**K18**	*A^1^A^2^*	nt. 428G/G (Se/Se)	A_1_ secretor	1.07	1.02	1.53
**K26**	*A^1^O^1^*	nt. 428G/A (Se/se)	A_1_ secretor	0.97	0.97	1.88
**K28**	*A^1^O^1v^*	nt. 428G/G (Se/Se)	A_1_ secretor	0.77	0.87	1.58
**Kd050**	*A^2^O^1^*	nt. 428G/A (Se/se)	A_2_ secretor	1.16	1.14	1.36
**K06**	*A^2^O^1v^*	nt. 428G/A (Se/se)	A_2_ secretor	1.00	0.95	3.38
**K17**	*A^2^O^1^*	nt. 428G/G (Se/Se)	A_2_ secretor	1.02	1.00	1.70
**K12**	*A^2^B*	nt. 428G/A (Se/se)	A_2_B secretor	1.25	1.05	1.17
**Kd011**	*BO^1^*	nt. 428A/A (se/se)	B non-secretor	1.03	0.94	1.17
**Kd006**	*BO^1^*	nt. 428G/A (Se/se)	B secretor	1.01	1.01	2.37
**K11**	*BO^1^*	nt. 428G/G (Se/Se)	B secretor	1.06	1.00	1.40
**K20**	*BO^1^*	nt. 428G/A (Se/se)	B secretor	1.05	0.97	2.39
**K25**	*BO^1^*	nt. 428G/G (Se/Se)	B secretor	1.22	1.08	1.31
**K13**	*O^1v^O^1v^*	nt. 428°/A (se/se)	O non-secretor	1.05	1.09	1.20
**K05**	*O^1^O^1v^*	nt. 428G/G (Se/Se)	O secretor	0.99	0.99	1.32
**K15**	*O^1^O^1v^*	nt. 428G/A (Se/se)	O secretor	0.94	0.91	1.32
**K19**	*O^1^O^1^*	nt. 428G/A (Se/se)	O secretor	1.08	1.03	1.19
**K21**	*O^1^O^2^*	nt. 428G/A (Se/se)	O secretor	0.98	0.97	1.50
**K22**	*O^1^O^1v^*	nt. 428G/G (Se/Se)	O secretor	1.13	1.00	1.42
**K27**	*O^1^O^1^*	nt. 428G/A (Se/se)	O secretor	0.94	1.14	1.17
**K29**	*O^1v^O^1v^*	nt. 428G/A (Se/se)	O secretor	0.94	0.96	1.72
**Average:**			**85% secretors**	**1.06**	**1.01**	**1.89**

The *ABO* [25], [26] and *FUT2* (secretor) genotype of MSCs was determined with PCR, the ABH histo-blood group phenotype predicted accordingly, and expression of ABH antigens was detected with flow cytometry. The relative fluorescence intensity (RFI) for binding of anti-A, -B, and –H was calculated by dividing the median fluorescence intensity (MFI) obtained for cells labeled with the corresponding antibody, by the MFI obtained for cells labeled with secondary antibody only. Abbreviations: *ABO*-ASP, *ABO* allele-specific primer PCR; *FUT2*-ASP, fucosyltransferase 2 allele-specific primer PCR (nucleotide 428 polymorphism).

### Clinical grade MSCs adsorb small quantities of ABO antigen from human AB serum and plasma, used for culture and infusion of cells, respectively

**Figure 2:**
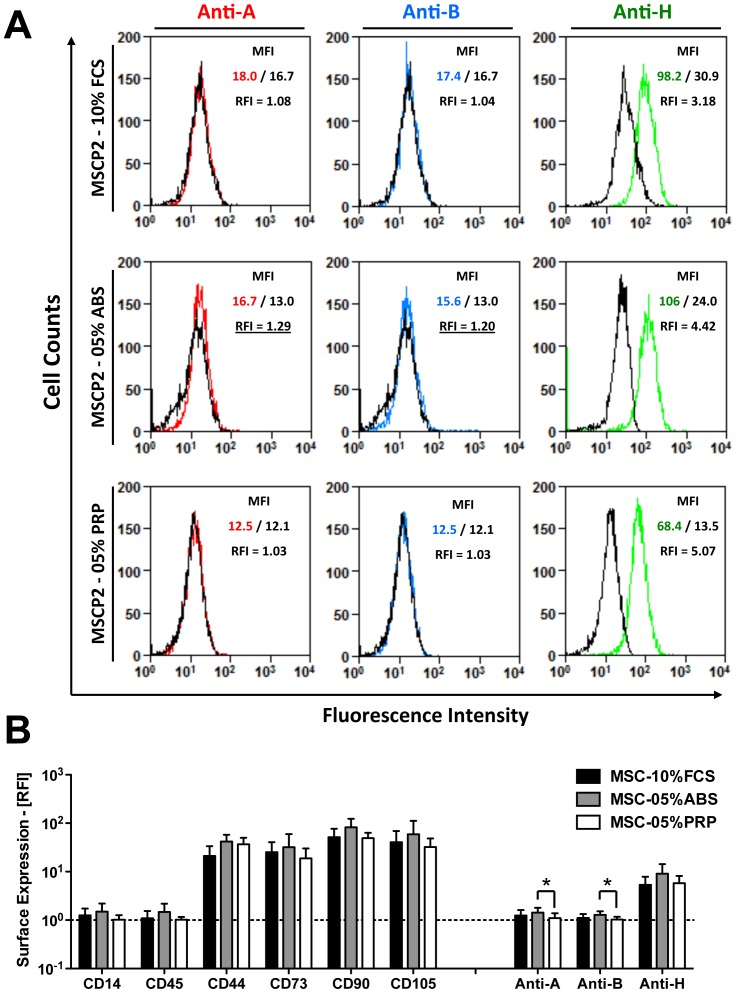
Adsorption of ABO antigens to MSCs from human AB culture serum. Clinical MSCs can be grown with three different culture supplements at our centers: 10% fetal calf serum (FCS), 5% human blood type AB serum (ABS), and 5% human platelet rich plasma (PRP), which may potentially contain antigens recognized by ABO-antibodies in patient blood [8]. We therefore used flow cytometry in order to detect the binding of anti-A/B antibodies to low passage MSCs (P1-4, n = 8 donors), expanded with either supplement. (**A**) Representative histogram overlays for binding of anti-A (red curve), anti-B (blue), and anti-H (green) antibodies to MSCs grown with different supplements, as compared to respective isotype controls (black curves). (**B**) Quantification of relative fluorescence intensity (RFI) compared to respective isotype controls, showing expression intensity of typical MSC surface antigens; Negative controls: CD45 (leukocyte common antigen) and CD14 (LPS receptor on myeloid cells); Positive controls: CD44 (Hyaluronic acid receptor), CD73 (Ecto-5’-nucelotidase), CD90 (Thy-1 antigen), CD105 (Endoglin); and binding of anti-A, anti-B, and anti-H antibodies. Mean ± SD, * *P* < 0.05.

We next wondered if ABO antigens could be adsorbed from the culture media used for cells isolation and expansion. Flow cytometry analysis of low passage MSCs cultured with 5% human ABS showed weak reactivity with anti-A/B antibodies (Figure 2A), which did not occur with cells grown in 5% human PRP or cell grown in 10% FCS (as documented earlier), while all cells expressed H antigen. Quantification of FACS data revealed that anti-A/B binding was significantly higher on cells grown in ABS media (P<0.05, Figure 2B), although positivity was generally very low for anti-A/B binding from culture medium, compared to expression of H antigen or MSC-typical surface markers CD44, CD73, CD90 and CD105.

**Figure 3:**
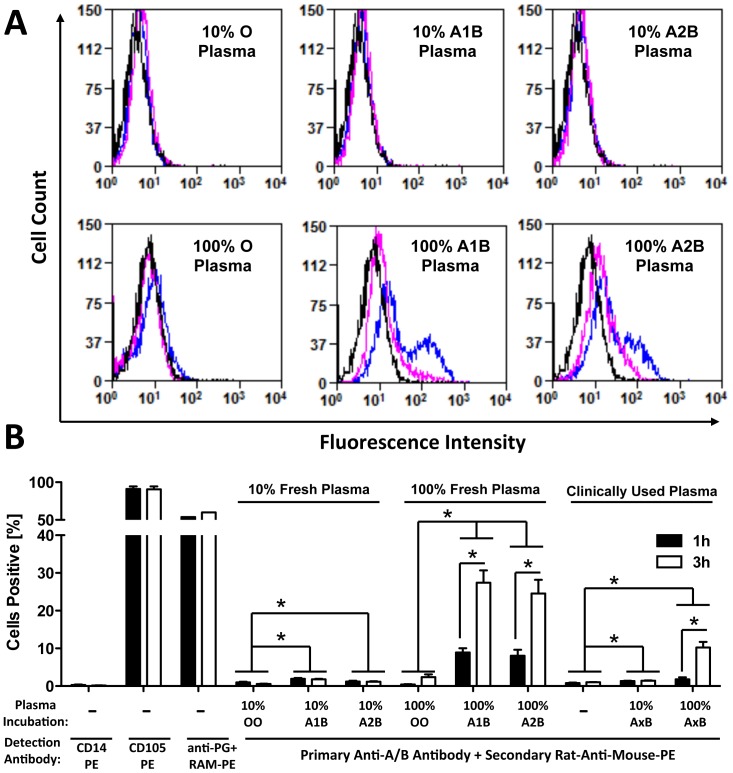
Adsorption of ABO antigens to MSCs from human AB plasma. MSCs are often washed and reconstituted in human AB plasma when prepared for systemic infusion. We therefore used flow cytometry, in order to detect potential binding of A/B-antigens to freshly thawed blood type O MSCs (n = 4) after incubation with human plasmas of different blood types (n = 3 each). (**A**) Histogram overlay for detection of soluble A/B-antigen binding from plasma to MSCs after a 1-hour (pink curve) or 3-hour (blue curve) incubation with 10% or 100% O, A_1_B, A_2_B, or clinical A_1/2_B plasma of unknown A1 or A2 subtype. Upon incubation, cells were washed with serum free media, to remove non-bound plasma components, and A/B-antigen binding was detected with primary mouse-anti-human A/B antibody, followed by incubation with secondary rat-anti-mouse-PE antibody (RAM-PE), and compared to binding of secondary antibody only to untreated cells (black curves). (**B**) Cells detected positive (%) after two different plasma adsorption times (1 *vs*. 3 hours). Five AB plasmas of unknowns A subtype, which were previously used for clinical MSC infusion (A_1/2_ B), were compared to O, A_1_B and A_2_B plasma. As controls, cells were labeled with CD14-PE (negative labeling control), CD105-PE (positive control), and anti-paragloboside (PG) + RAM-PE (secondary antibody labeling control), or anti-A/B + RAM-PE, to detect A/B-antigen binding, as indicated below the figure. Mean±SD, * *P* < 0.05.

Clinical MSCs are often washed and reconstituted in PBS buffer containing 10% fresh ABP for intravenous infusion. The ABP may contain varying levels of immunogenic soluble ABO antigens. In analogy to the culture serum test, we wondered if these soluble ABO antigens in AB plasma could be adsorbed by MSCs, and consequently recognized by the patients’ anti-A/B antibodies, leading to immune destruction. In a mechanistic approach, we incubated blood group O MSCs in 10% or 100% fresh plasma of known blood groups or clinically used plasmas (A_1/2_B) of unknown A subgroup (Figure 3A). Flow cytometry analysis detected a weak shift for binding of anti-A/B after incubation with 10% plasma and a strong shift after incubation with 100% A_1_B or A_2_B plasma, but not with O plasma. The signal shift increased after 3 hours of incubation (blue line), compared to the 1-hour incubation time point (pink line). Cell quantification confirmed a weak but significant increase for detection of anti-A/B binding upon incubation with 10% A_1_B, A_2_B, and A_1/2_B plasma, as compared to O plasma (P<0.05, Figure 3B). We furthermore found a 3-fold increase of anti-A/B-binding to cells treated with 100% A_1_B, A_2_B, and A_1/2_B plasma after 3 hours of incubation (P<0.05), compared to 1 hour of incubation, suggesting a time- and dose-dependent adsorption of A and B antigens. Positivity of labeling controls for detection of CD105 (<95% positive) and paragloboside (>60% positive) did not change with time, whilst positivity for negative control CD14 was negligible over the course of incubation.

### Washing of MSCs with ABP-containing buffer triggers stronger IBMIR responses *in vitro* and is associated with a trend for lower response in blood group O recipients *in vivo*

**Figure 4:**
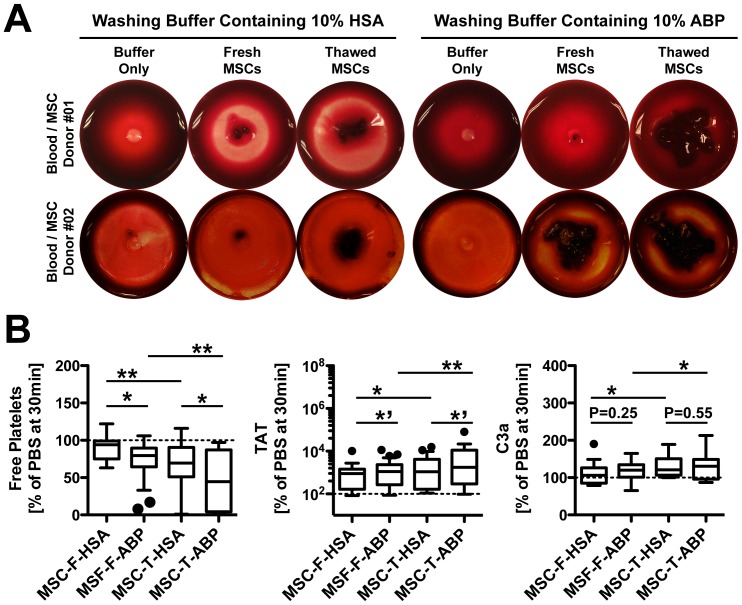
Whole blood exposure of MSCs reconstituted in different supplements. Freshly harvested or freeze-thawed MSCs (15,000 cells per ml, n = 18), washed and reconstituted in buffer containing 10% human AB plasma (ABP) or 10% human serum albumin (HSA), were tested for triggering of the instant blood mediated inflammatory reaction (IBMIR) by exposing them to non-anticoagulated whole blood in the chandler blood loop model. (**A**) Representative photographs of clot formation after a 60-min incubation of blood with MSCs, or buffer as negative control. (**B**) Percentage (% relative to PBS, 30 minutes time point) of coagulation and complement activation markers after treatment of blood with fresh or thawed MSCs (MSC-F or MSC-T, respectively, n = 18 each): free platelets, and ELISA quantification of thrombin-anti-thrombin complex (TAT), and complement C3 activation fragment a (C3a). Box plot whiskers Tukey: *’ *P*<0.1, * *P*<0.05, and ** *P*<0.01.

To verify if MSCs react differently with human blood – in the presence or absence of soluble ABO antigens in washing buffer – we washed and reconstituted the cells in buffer containing either 10% ABP or HSA and subsequently exposed them to blood from healthy, immunocompentent blood group O donors. Visual clot evaluation showed typical donor variation, but a tendency for stronger clot formation with cells resuspended in 10% ABP, particularly for freshly thawed cells, as predominantly used in the clinic (Figure 4A). Cells resuspended in buffer containing ABP elicited a significantly stronger activation of the clotting cascade, as indicated by stronger reduction of free platelets and increased formation of thrombin, which were both stronger for thawed cells (P<0.1, P<0.05 and P<0.01, Figure 4B). ABP-exposed MSCs showed a trend for increased formation of complement activation marker C3a, but this was only significantly increased for thawed cells (P<0.05).

We next analyzed the anti-A/B agglutination titers in patient plasma of MSC recipients at a median 3.5 days prior (n = 19) and 14 days post MSC-infusion (n = 10). Anti-A/B titers at MSC infusion were generally low in our patient cohort (Table 2), and did not show a significant change 14 days post MSC infusion (data not shown). As expected, the highest levels of anti-A/B titers were found in blood group O recipients (n = 9, mean IgM/IgG titer of anti-A 1:4/1:10 and anti-B 1:6/1:11, respectively), whilst blood group A/B/AB recipients had almost no detectable anti-A/B titers (n = 10, mean IgM/IgG titer of anti-A undetectable and anti-B 1:1/1:2, respectively). No correlation between the very low MSC recipient anti-A/B agglutination titers and clinical response to MSC treatment was found (P>0.05, Figure S2A and S2B). Evaluation of clinical responses in patients treated with MSCs showed no difference in response for recipients with blood group O before HSCT procedure, compared to patients with original blood group A, B, or AB (Table 3), when combining ABP-exposed and HSA-exposed MSC products. Interestingly, when performing analysis on patients infused with ABP-exposed MSC products (n = 70), we found a trend for a lower clinical response in O recipients compared to blood group A, B, or AB recipients (P = 0.09, 58% vs. 77%, Table S2).

**Table 2:** HSCT patient ABO antibody titers at the time of MSC infusion.

MSC Recipient		Blood Group		Agglutination Titers		MSC Treatment	
Subject Code	Sex/Age (years)	HSC R/D	MSC Donor	Anti-A IgM/IgG	Anti-BIgM/IgG	Treatment Indication	Clinical Response
910	M/45	AB/A	O	0/0	0/0	GvHD	SD
917	F/61	AB/A	B	0/0	0/0	GvHD	PD
1007	M/59	AB/A	A	0/0	0/0	GvHD	CR
1071	M/48	AB/A	AB	0/0	0/0	GvHD	PD
1068	F/34	A/A	O	0/0	8/8	HC	PR
1080	F/56	A/A	B	0/0	2/2	GvHD	CR
1101	M/55	A/A	AB	0/0	2/2	GvHD	PD
1115	M/55	A/A	O	0/0	1/1	GvHD	CR
1139	M/02	A/A	AB	0/0	0/4	GvHD	CR
1311	F/39	A/B	A	0/0	16/16	GvHD	CR
1082	F/64	A/O	B	0/0	0/1	GvHD	PR
1271	M/36	A/O	A	0/0	16/16	GvHD	PR
1048	M/48	B/O	B	1/8	0/0	HC	PR
1141	M/65	B/O	O	0/4	4/4	GvHD	PD
1163	M/31	O/B	A	0/0	1/4	GvHD	CR
1084	M/35	O/O	O	0/4	1/4	GvHD	PD
1090	F/01	O/O	A	0/8	0/2	GvHD	CR
1098	M/14	O/O	A	32/64	32/64	HC	CR
1131	F/53	O/O	O	0/4	1/2	GvHD	PD

Abbreviations: MSC, mesenchymal stromal cell; HSC, hematopoietic stem cell; R/D, donor/recipient; Anti-A or anti-B IgM/IgG, agglutination titers of anti blood group antigen A or B antibodies of immunoglobulin M or G type; GvHD, graft versus host disease; and clinical response to MSC treatment expressed as: CR, complete response; PR, partial response; SD, stable disease; and PD, progressive disease.

**Table 3:** Evaluation ABO-related clinical response to MSCs.

Patient blood type before HSCT:	O	A, B, AB	P-value
**Patient sex:** male/female	**23/08**	**29/13**	**0.6312**
**Patient age:** median (range)	**42 (1.0 – 65)**	**45 (1.0 – 68)**	**0.4055**
**Patient age:** children/adults	**11/20**	**09/33**	**0.1832**
**Indication for MSC treatment:**			**0.3044**
Graft-versus-host disease	26	31	
Tissue injury after HSCT	5	11	
**MSC donor sex:** male/female	**24/24**	**29/28**	**0.9286**
**MSC donor age:** median (range)	**37 (24 – 66)**	**33 (6.0– 66)**	**0.0637**
**MSC cell passage:** median (range)	**2.6 (1.0 – 4.0)**	**2.6 (1.0 – 3.0)**	**0.9204**
**MSC cell dose:** median x10^6^/kg (range)	**1.7 (0.7 – 4.2)**	**1.7 (0.7 – 3.0)**	**0.6661**
**MSC HLA match with recipient:**	**07/48 (15%)**	**08/57 (14%)**	**0.9363**
Third party unrelated donor	41	49	
HLA-identical sibling or related	7	8	
**Patient response to treatment:**	**33/48 (69%)**	**38/57 (67%)**	**0.8202**
Complete and partial responders	33	38	
Stable and progressive disease	15	19	

Patient characteristics and evaluation of clinical response to individual MSC-infusions in patients undergoing HSCT (Stockholm, n = 70; and Leiden, n = 35 MSC infusions). Blood type O (containing highest titers of both anti-A/B antibodies) was compared to blood type A, B, and AB (blood containing anti-B, anti-A, or no anti-A/B antibodies, respectively). Abbreviations: HSCT, hematopoietic stem cell transplantation; MSC, mesenchymal stromal cell; BG, blood group; HLA, human leukocyte antigen. Statistics: P-value is calculated using Mann-Whitney rank-sum test (for continuous variables), Fisher’s exact t-test (comparing two categorical variables), or Chi^2^-test (comparing more than two categorical variables).

## Discussion

Interpretation of the clinical relevance of ABO blood groups for clinical MSC application is challenging, due to the many variables that need to be considered. We have designed this study as a prototype analysis, to be used for comparable analysis in other treatment indications, where therapeutic MSCs or other MSC-like products are given to more immunocompentent patients. Patients that have undergone HSCT are strongly immunocompromised. Limited efficacy of MSC treatment due to immunogenic blood groups may be more difficult to document. However, this well characterized patient group could serve as an example, for maximizing the therapeutic efficacy of these cells in various treatment approaches.

Depending on the therapeutic approach and its underlying limitations, different major choices in manufacturing therapeutic MSCs have to be considered. The most common cell source is cryo-banked third party MSCs cultured for 2–4 passages in media containing FCS. Cell handling directly before infusion is also important, as different centers have developed specific routines for cell washing and infusion [4], affecting cell function [32]. MSCs are often washed and infused in buffer containing supplements such as ABP, HSA and low dose heparin. The suspension medium was shown to influence the interaction of MSCs with endothelium and pulmonary toxicity after infusion [32]. Interpretation of ABO status for HSCT-transplant patients is multifactorial. One needs to consider the original blood group of the HSCT recipient and its possible change after obtaining the HSCT graft, and the impact of HSCT related procedures, such as transfusions, on circulating antibody levels at the time of MSC infusion. Here we present a stepwise analysis on the possible impact of ABO blood groups for the outcome of MSC therapy.

### Presence of ABO antigens in native clinical grade MSCs

As outlined earlier, intrinsic antigen expression in native clinical grade MSCs and its change upon encounter of stimulatory or differentiating events *in vivo* may affect outcome. It has previously been suggested that ABO status is of minor importance for MSC therapy since MSCs do not express or adsorb ABO antigens on their surface [18], [19]. Our present study found, that although the *ABO* promoter region in MSCs is methylated to a similarly low degree as the housekeeping gene GAPDH, the cells do not express *ABO* transcripts under resting or stimulating conditions. There are many possible explanations for a lack of *ABO* transcript expression in MSCs, such as repressive histone marks, nuclear localization in repressive nuclear compartments, a lack of transcription factors regulating this particular gene in these particular cells, or possibly the presence of regulatory micro-RNAs. The ABH antigens, carried by four main types of carrier carbohydrate chains, are typically found on erythrocytes, but are also present at high density in epithelial and endothelial cells [33]. For both A and H determinants, branched or un-branched type 2 chains exist, with the unbranched type 2 chain (classical i antigen) being predominant in fetal/newborn cells, while the branched form (classical I antigen) is predominant in adult cells. A recent publication by Hirvonen et al. [34] highlights that particularly umbilical cord blood derived MSCs show “immature” cell-specific expression of the blood group i epitope (linear poly-*N*-acetyllactosamine chain), which was not found in differentiated cells. Further in-depth studies on the “glycomics” of MSCs may therefore be of interest in “mesenchymal stem cell” phenotyping and to evaluate MSCs differentiation stages [35].

### Contamination of clinical MSCs with ABO antigens from supplements and relevance of ABO status for clinical application of therapeutic MSCs

MSCs show a hypo-immunogenic profile, which allows their transplantation across HLA barriers, although autologous cells can be used in many clinical settings. Importantly, previous studies indicate that the therapeutic efficacy of MSCs could potentially be compromised by immunogenic substances derived from culture supplements used in MSC production, such as xeno-antigens derived from FCS [13]. Spees and coworkers found that 7 to 30 mg of FCS proteins can be incorporated in a typical dose of these cells (100 million cells), but FCS antigen contamination can be reduced to less than 100 ng (100.000-fold reduction) by washing with human serum. Feasible alternatives to FCS, such as pooled human ABS, HPL and PRP have therefore been established in the past decade [14]–[17]_ENREF_14. Nonetheless, also human substitutes must be handled with care, due to inter-individual differences, such as donor blood group and HLA type, and be prepared accordingly, to minimize any adverse reactions [16]. In agreement with Schäfer et al. [19], we found that MSCs do at best only display minimal amounts of A and B antigens on their surface, after culture in media containing ABS, highlighting the safety of ABS for MSC expansion, although supply is limited due to the scarcity of group AB donors (only less than 5% of blood donors are of AB group). We found no reactivity with anti-A/B antibodies after culture in media containing FCS or PRP, supporting the choice of abundant PRP for expansion of MSCs [14]–[17]. Importantly, incubation of MSCs with fresh ABP, which is used for washing and cell infusion, lead to a clear dose and time-dependent increase in anti-A/B binding, which did not occur with plasma from group O donors, raising the question if the amount of adsorbed ABO antigen on MSCs surface is sufficient to elicit an adverse whole blood response?

*In vitro* exposure to blood from immunocompentent blood group O donors, displaying high titers of anti-A/B titers of ABO antibodies [21], lead to an increased triggering of the instant blood mediated inflammatory reaction (IBMIR). Triggering of IBMIR was most evident when using thawed cells resuspended in ABP buffer, as opposed to HSA buffer, strongly advocating for replacement of poorly defined ABP by GMP-compliant HSA, to minimize any unnecessary adverse reactions [7], [36]. This is of particular importance when giving MSCs to immunocompentent patient groups, such as in treatment of type-1 diabetes or multiple sclerosis. Could preformed naturally-occurring anti-A/B antibodies have affected MSCs therapeutic efficiency in HSCT patients? These antibodies can potentially mediate graft rejection [8], [37]. Particularly patients with higher titers of anti-A/B would be at risk to reject ABO-antigen-contaminated MSCs. It is known from solid organ transplantation that predominantly adult blood group O recipients are a risk group for early allograft rejection, due to the presence of higher levels of anti-A/B IgG antibodies [21]. We generally found only very low agglutination titers of anti-A/B in our patient cohort and no correlation with direct clinical outcome. The conditioning prior to HSCT and prevention and treatment of established GvHD likely account for the low antibody titers and possibly confounds our analysis. When evaluating the clinical outcome with respect to MSC recipients’ blood group before or after HSCT (O vs. A, B, and AB) we found a trend for lower response in blood group O recipients. It appears that the quantities of anti-A/B immunoglobulin found in these strongly immunocompromised patients are insufficient to elicit a relevant response towards therapeutic MSCs when used for the treatment of HSCT disorders, such as acute GvHD and hemorrhagic cystitis, but immunocompetent patients could potentially reject MSCs washed with immunogenic ABP.

## Conclusion and Summary

MSCs do not inherently express ABO antigens, but can potentially adsorb significant amounts of immunogenic antigens with cross reactivity to anti-A/B from undefined human serum/plasma, used for cell production and infusion, respectively. We here show, that MSCs do not adsorb immunogenic ABO antigens from culture media containing FCS or human substitutes such as ABS and PRP as supplements, but MSC adsorbed antigen from the fresh ABP used for washing and cell infusion. Blood exposure of cells resuspended in buffer containing ABP elicited stronger triggering of IBMIR *in vitro*. We found a trend for worse clinical outcome in blood group O recipients receiving ABP-exposed MSCs, who displayed higher amounts of circulating ABO antibodies. We therefore suggest exchanging ABP by more defined, non-immunogenic, commercially available HSA, particularly when giving cells to immunocompetent patients, to eliminate this unnecessary factor of uncertainty and to maximize the cell product quality and functionality *in vivo*.

## Supporting Information

Figure S1 **ABO antigen surface expression in clinical grade MSCs.** Flow cytometry was used to detect the expression of ABH antigens on freshly thawed low-passage clinical grade MSCs. **(A)** Representative histogram plots for 3 MSCs (Code K7: A-secretor, K25: B-secretor, and K15: O-secretor) labeled with anti-A (left, red line), anti-B (middle, blue), anti-H (right panel, green), are shown compared to MSCs labeled with secondary antibody only (black line). The median fluorescence intensity (MFI) obtained with respective test labeling (red, green, and blue), or labeling with secondary antibody only (black), is depicted in the histograms, and the relative fluorescence intensity (RFI) was calculated by dividing the MFI of anti-ABH-labeled cells, by the MFI obtained with secondary antibody only. **(B)** Summary for detection of ABH or paragloboside (PG) carbohydrate antigen on the cell surface of blood type A (black), type B (grey), and type O (white) MSCs. The cells were grouped according to their putative secretor (full symbols) or non-secretor status (shaded symbols), which was determined by *FUT2* (secretor) genotyping, as summarized in Table 1. Data are expressed as RFI and presented as means ± SD.
(TIF)

Figure S2 **Potential impact of recipient anti-A/B titers for clinical response.** Patient evaluation of responders (CR, complete response, and PR, partial responder) and non-responders (SD, stable disease, and PD, progressive disease) to MSC treatment shows no significant differences when comparing: **(A)** Agglutination titers of all ABO antibodies (combining Anti-A + Anti-B, and IgG + IgM; P = 0.3154), or **(B)** Agglutination titers of ABO antibodies separating anti-A/B immunoglobulin G (IgG; P = 0.2514) and IgM (P = 0.6036).
(TIF)

Table S1 **Antibodies and reagents used for immunostaining.**
(DOCX)

Table S2 **Evaluation ABO-related clinical response to ABP-exposed MSCs.** Patient characteristics and evaluation of clinical response to ABP-exposed MSCs. Blood type O (containing highest titers of both anti-A/B antibodies) was compared to blood type A, B, and AB (anti-B, anti-A, or no anti-A/B antibodies, respectively). Abbreviations: HSCT, hematopoietic stem cell transplantation; MSC, mesenchymal stromal cell; BG, blood group; HLA, human leukocyte antigen. Statistics: P-value is calculated using Mann-Whitney rank-sum test (for continuous variables), Fisher’s exact t-test (comparing two categorical variables), or Chi^2^-test (comparing more than two categorical variables).
(DOCX)
