# Supplementary figures and images for: Do ABO Blood Group Antigens Hamper the Therapeutic Efficacy of Mesenchymal Stromal Cells?
Source: PLoS One. 2014 Jan 13;9(1):e85040. doi: 10.1371/journal.pone.0085040 (PMC3890285; doi:10.1371/journal.pone.0085040)

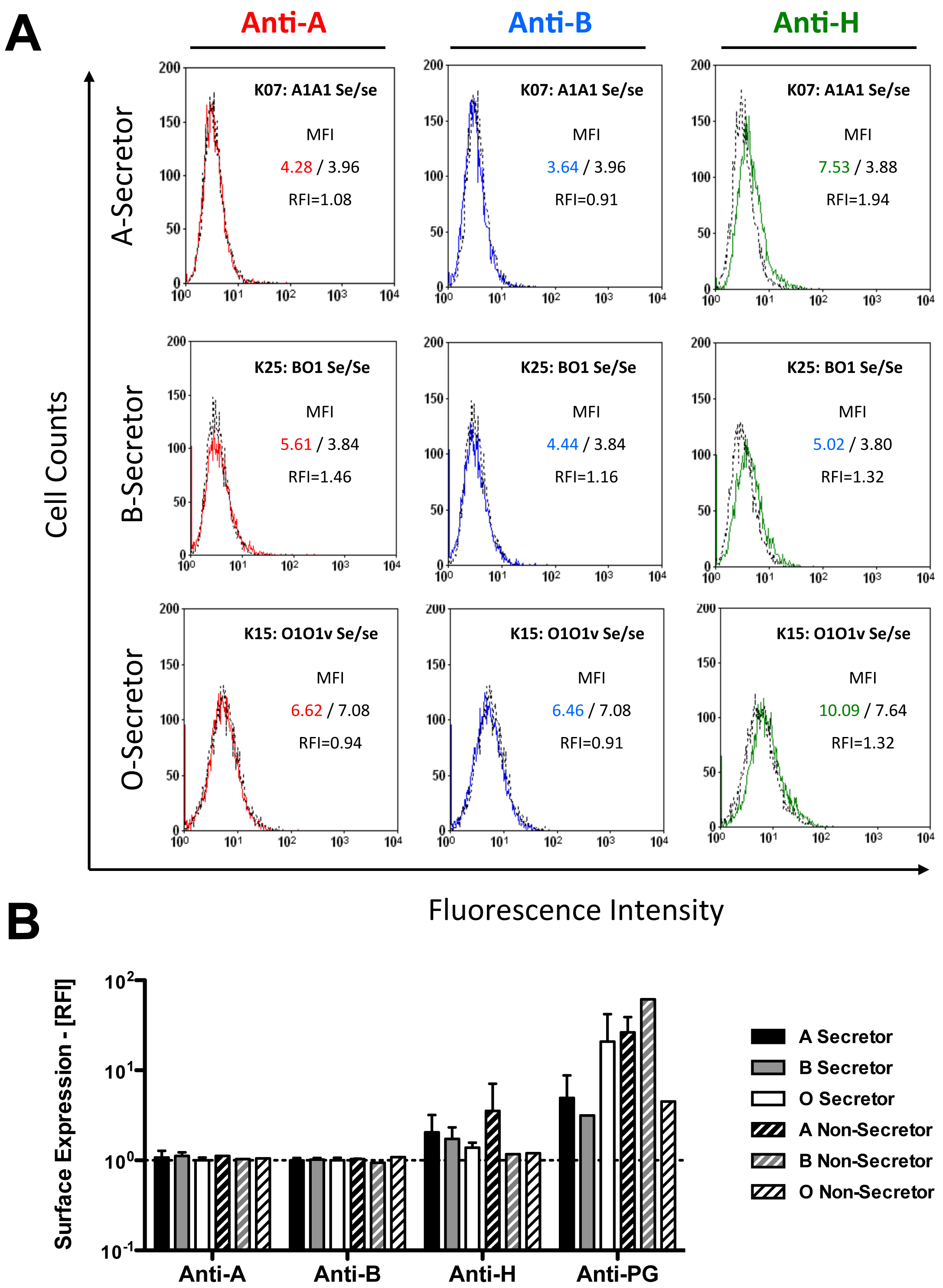

Supplement: Figure S1 — ABO antigen surface expression in clinical grade MSCs. Flow cytometry was used to detect the expression of ABH antigens on freshly thawed low-passage clinical grade MSCs. (A) Representative histogram plots for 3 MSCs (Code K7: A-secretor, K25: B-secretor, and K15: O-secretor) labeled with anti-A (left, red line), anti-B (middle, blue), anti-H (right panel, green), are shown compared to MSCs labeled with secondary antibody only (black line). The median fluorescence intensity (MFI) obtained with respective test labeling (red, green, and blue), or labeling with secondary antibody only (black), is depicted in the histograms, and the relative fluorescence intensity (RFI) was calculated by dividing the MFI of anti-ABH-labeled cells, by the MFI obtained with secondary antibody only. (B) Summary for detection of ABH or paragloboside (PG) carbohydrate antigen on the cell surface of blood type A (black), type B (grey), and type O (white) MSCs. The cells were grouped according to their putative secretor (full symbols) or non-secretor status (shaded symbols), which was determined by FUT2 (secretor) genotyping, as summarized in Table 1. Data are expressed as RFI and presented as means ± SD. (TIF) [file pone.0085040.s001.tif]

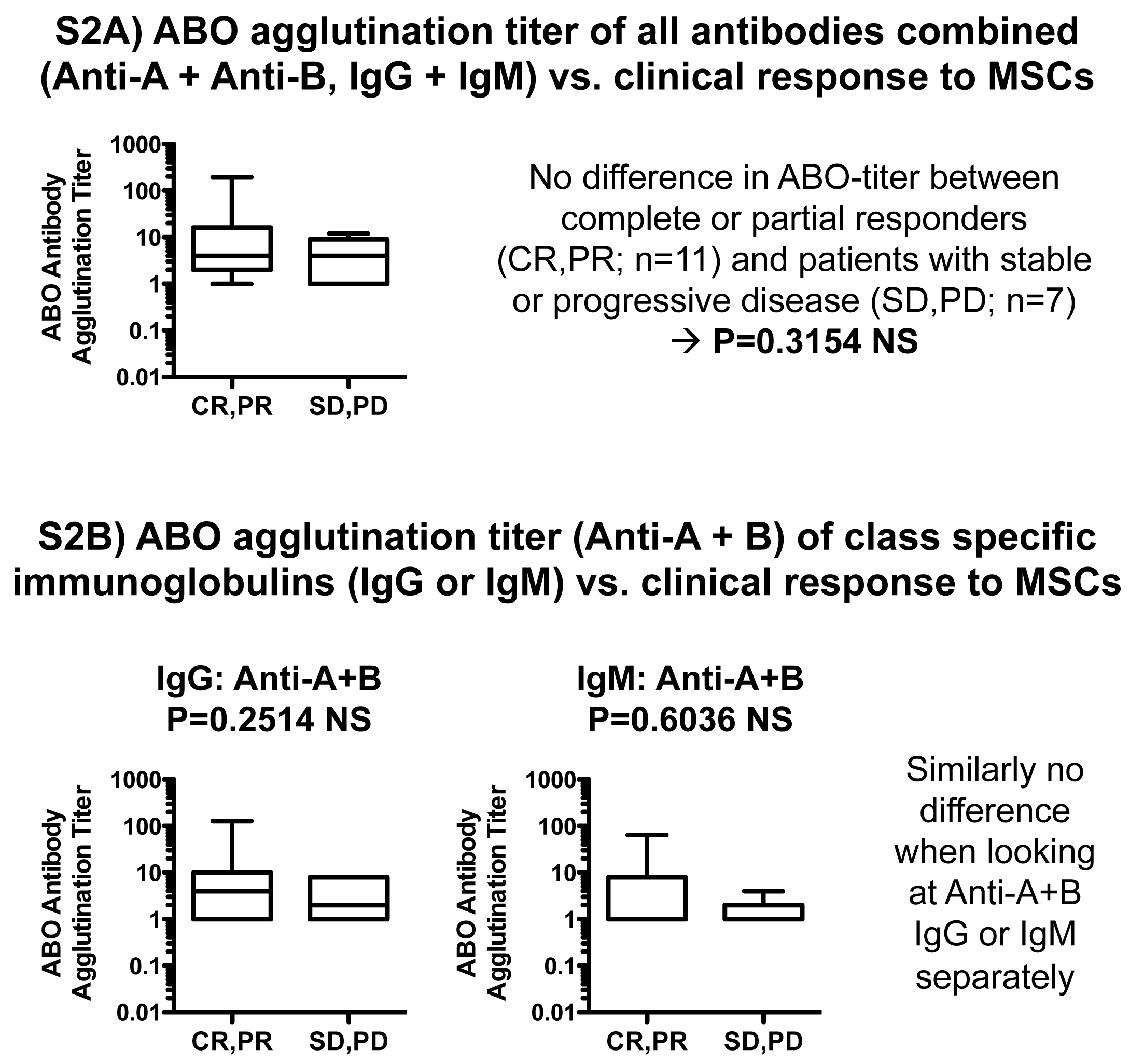

Supplement: Figure S2 — Potential impact of recipient anti-A/B titers for clinical response. Patient evaluation of responders (CR, complete response, and PR, partial responder) and non-responders (SD, stable disease, and PD, progressive disease) to MSC treatment shows no significant differences when comparing: (A) Agglutination titers of all ABO antibodies (combining Anti-A + Anti-B, and IgG + IgM; P = 0.3154), or (B) Agglutination titers of ABO antibodies separating anti-A/B immunoglobulin G (IgG; P = 0.2514) and IgM (P = 0.6036). (TIF) [file pone.0085040.s002.tif]
