# Supplementary material for: Do ABO Blood Group Antigens Hamper the Therapeutic Efficacy of Mesenchymal Stromal Cells?
Source: PLoS One. 2014 Jan 13;9(1):e85040. doi: 10.1371/journal.pone.0085040 (PMC3890285; doi:10.1371/journal.pone.0085040)
Supplement: Table S1 — Antibodies and reagents used for immunostaining. (DOCX) [file pone.0085040.s003.docx]

**Table S1: Antibodies and reagents used for immunostaining.**

| **Antibody** | **Host** | **Target / Antigen** | **Code** | **Company** |
| --- | --- | --- | --- | --- |
| **IgG1/2a (FITC/PE)** | Mouse | Mouse IgG1/2a Isotype  (FITC/PE conjugated) | #342409 | Becton Dickinson  (CA, USA) |
| **CD14/45 (FITC/PE)** | Mouse | Anti-human CD45/PE  (FITC/PE conjugated) | #342408 | Becton Dickinson  (CA, USA) |
| **CD44**  **(FITC)** | Mouse | Anti-human CD44  (FITC conjugated) | #338804 | Bio Legends  (USA) |
| **CD73**  **(PE)** | Mouse | Anti-human Ecto-5’  Nucleotidase (PE) | #550257 | Becton Dickinson  (CA, USA) |
| **CD90**  **(FITC)** | Mouse | Anti-human Thy-1  (FITC conjugated) | #555595 | Becton Dickinson  (CA, USA) |
| **CD105**  **(PE)** | Mouse | Anti-human Endoglin  (PE conjugated) | #326040 | Ancell  (Bayport, USA) |
| **Anti-H** | Mouse | Anti-human blood group H  Epitope (FITC conjugated) | Clone  BRIC 231 | BITS/IBGRL  (Bristol, UK) |
| **Anti-A** | Mouse | Anti-human blood group A  Epitope (unconjugated Ab) | Clone  ES-15 | Serologicals  (West Lothian, UK) |
| **Anti-B** | Mouse | Anti-human blood group B  Epitope (unconjugated Ab) | Clone  9621A8 | Diagast  (France) |
| **Anti-PG** | Mouse | Anti-human paragloboside  Epitope (unconjugated Ab) | Clone  1B2 | Gift from Henrik Clausen (Denmark) |
| **Rat anti Mouse** | Rat | PE-labeled 2^nd^ rat-anti-mouse Ig kappa light chain | #562021 | Becton Dickinson  (CA, USA) |
