# Supplementary material for: Do ABO Blood Group Antigens Hamper the Therapeutic Efficacy of Mesenchymal Stromal Cells?
Source: PLoS One. 2014 Jan 13;9(1):e85040. doi: 10.1371/journal.pone.0085040 (PMC3890285; doi:10.1371/journal.pone.0085040)
Supplement: Table S2 — Evaluation ABO-related clinical response to ABP-exposed MSCs. Patient characteristics and evaluation of clinical response to ABP-exposed MSCs. Blood type O (containing highest titers of both anti-A/B antibodies) was compared to blood type A, B, and AB (anti-B, anti-A, or no anti-A/B antibodies, respectively). Abbreviations: HSCT, hematopoietic stem cell transplantation; MSC, mesenchymal stromal cell; BG, blood group; HLA, human leukocyte antigen. Statistics: P-value is calculated using Mann-Whitney rank-sum test (for continuous variables), Fisher’s exact t-test (comparing two categorical variables), or Chi2-test (comparing more than two categorical variables). (DOCX) [file pone.0085040.s004.docx]

**Table S2: Evaluation ABO-related clinical response to ABP-exposed MSCs.**

| **Patient blood type before HSCT:** | **O** | **A, B, AB** | **P-value** |
| --- | --- | --- | --- |
| **Patient sex:** male / female | **14 / 04** | **22 / 08** | **0.7306** |
| **Patient age:** median (range) | **40 (1.0 – 65)** | **47 (1.0 – 67)** | **0.3539** |
| **Patient age:** children / adults | **07 / 11** | **05 / 25** | **0.0852** |
| **Indication for MSC treatment:** |  |  | **0.5271** |
| Graft-versus-host disease | 13 | 19 |  |
| Tissue injury after HSCT | 5 | 11 |  |
| **MSC donor sex:** male / female | **15 / 16** | **20 / 19** | **0.8098** |
| **MSC donor age:** median (range) | **38 (24 – 66)** | **33 (6.0 – 66)** | **0.0520** |
| **MSC cell passage:** median (range) | **2.8 (1.0 – 4.0)** | **2.7 (1.0 – 3.0)** | **0.2641** |
| **MSC cell dose:** median x10^6^/kg (range) | **1.7 (0.7 – 4.2)** | **1.7 (0.7 – 3.0)** | **0.6653** |
| **MSC HLA match with recipient:** | **07 / 31 (23%)** | **06 / 39 (15%)** | **0.4419** |
| Third party unrelated donor | 24 | 33 |  |
| HLA-identical sibling or related | 7 | 6 |  |
| **Patient response to treatment:** | **18 / 31 (58%)** | **30 / 39 (77%)** | **0.0914** |
| Complete and partial responders | 18 | 30 |  |
| Stable and progressive disease | 13 | 09 |  |

Patient characteristics and evaluation of clinical response to ABP-exposed MSCs. Blood type O (containing highest titers of both anti-A/B antibodies) was compared to blood type A, B, and AB (anti-B, anti-A, or no anti-A/B antibodies, respectively). Abbreviations: HSCT, hematopoietic stem cell transplantation; MSC, mesenchymal stromal cell; BG, blood group; HLA, human leukocyte antigen. Statistics: P-value is calculated using Mann-Whitney rank-sum test (for continuous variables), Fisher’s exact t-test (comparing two categorical variables), or Chi^2^-test (comparing more than two categorical variables).
